# Prevalence of knee arthroplasty in the state of São Paulo between 2003 and 2010: Prevalência de artroplastia de joelho no estado de São Paulo entre 2003 e 2010

**DOI:** 10.1590/1516-3180.2016.0111300616

**Published:** 2016-09-26

**Authors:** Rogério Teixeira de Carvalho, Jonny Chaves Lima Canté, Juliana Hoss Silva Lima, Luiz Alberto Barbosa Tavares, Marcelo Itiro Takano, Fernando Gomes Tavares

**Affiliations:** I MD. Attending Physician in the Knee Group, Orthopedics and Traumatology Service, Hospital do Servidor Público do Estado de São Paulo, São Paulo (SP), Brazil.; II MD. Fellow in the Knee Group, Orthopedics and Traumatology Service, Hospital do Servidor Público do Estado de São Paulo, São Paulo (SP), Brazil.; III MSc. Statistician, Orthopedics and Traumatology Service, Hospital do Servidor Público do Estado de São Paulo, São Paulo (SP), Brazil.; IV MD. Fellow in the Pediatric Orthopedics Group, Orthopedics and Traumatology Service, Hospital do Servidor Público do Estado de São Paulo, São Paulo (SP), Brazil.; V MD. Attending Physician in the Hip Group, Orthopedics and Traumatology Service, Hospital do Servidor Público do Estado de São Paulo, São Paulo (SP), Brazil.

**Keywords:** Knee, Osteoarthritis, Prevalence, Arthroplasty, Hospitals, Joelho, Osteoartrose, Prevalência, Artroplastia, Hospitais

## Abstract

**CONTEXT AND OBJECTIVE:**

The volume of knee arthroplasty procedures has increased over the last decade. The aim of this study was to estimate the frequency of these procedures performed within the public healthcare system of the state of São Paulo between 2003 and 2010.

**DESIGN AND SETTING:**

Cross-sectional study conducted in the state of São Paulo by researchers at Hospital do Servidor Público do Estado de São Paulo.

**METHODS:**

A sample of 10,952 patients (7,891 females and 3,061 males) who underwent primary total knee arthroplasty (TKA) and revision of total knee arthroplasty (RTKA) in the state of São Paulo between 2003 and 2010 was evaluated. The patients were cataloged using the public healthcare service's TABNET software. All of the patients presented primary osteoarthritis of the knee. The variables of gender, number of primary TKA procedures and number of RTKA procedures were evaluated.

**RESULTS:**

A total of 10,952 TKA procedures were performed (annual average of 1369), of which 9,271 (85%) were TKA and 1,681 (15%), RTKA. Of the TKA procedures, 72% were carried out on females (P < 0.0001), while 70% of the RTKA procedures were on females (P < 0.0001). The average ratio of TKA to RTKA was 5.5:1 (P < 0.0001); the ratios in 2003 and 2010 were 9.0:1 and 4.4:1 (P < 0.0001), respectively.

**CONCLUSION:**

The number and frequency of TKA and RTKA procedures increased in the state of São Paulo between 2003 and 2010. This increase was relatively greater in RTKA than in TKA and was predominantly in female patients.

## RESUMO

**CONTEXTO E OBJETIVO:**

O volume das artroplastias de joelho tem crescido na última década. O objetivo deste estudo foi estimar a frequência desses procedimentos realizados no Sistema Público de Saúde (SUS) do Estado de São Paulo no período de 2003 a 2010.

**TIPO DE ESTUDO E LOCAL:**

Estudo transversal conduzido por pesquisadores do Hospital do Servidor Público Estadual.

**MÉTODOS:**

Foi avaliada uma amostra de 10.952 pacientes (7.891 mulheres e 3.061 homens) submetidos a artroplastia primária total de joelho (ATJ) e a revisão de artroplastia total de joelho (RATJ) no estado de São Paulo entre 2003 e 2010. Os pacientes foram catalogados por meio do programa TABNET do SUS. Todos os pacientes eram portadores de osteoartrite primária do joelho. As variáveis analisadas foram: gênero, número de ATJs e número de RATJs.

**RESULTADOS:**

No total, 10.592 ATJs foram realizadas (média anual de 1.369 procedimentos), com 9.271 (85%) ATJs e 1.691 (15%) RATJ. Das ATJ, 72% ocorreram em mulheres (P < 0.0001), enquanto 70% das RATJs foram em mulheres (P < 0.0001). A relação média de ATJ para RATJ foi de 5.5:1 (P < 0.0001), com proporção em 2003 e 2010 de 9.0:1 e 4.4:1 (P < 0.0001), respectivamente.

**CONCLUSÃO:**

O número e a prevalência das ATJs e RATJs aumentaram no estado de São Paulo no período de 2003 a 2010. Essa elevação foi relativamente maior nas RATJs quando comparadas com as ATJs e ocorreu predominantemente nos pacientes do sexo feminino.

## INTRODUCTION

It has been estimated that 4% of the Brazilian population over the age of 60 years suffer from osteoarthritis (OA), and the knee is the joint that is second most commonly affected, with 37% of the cases.^1^ The population of the state of São Paulo in 2000 was approximately 37 million inhabitants, and the population over the age of 60 represented 8.9% of this total.^2^ By 2012, this percentage had increased to 11.5%, out of a total of 41 million people living in São Paulo.^2^

Over the last few decades, the rate of increase in the number of elderly people has outstripped the birthrate,^3^ and there has been an expansion in the absolute numbers of cases of injury due to OA of the knee.^4^ One of the therapeutic options for advanced symptomatic OA is total knee arthroplasty (TKA), which has shown a long-term success rate of 85% for relieving pain and improving function.^4^^,^^5^^,^^6^^,^^7^^,^^8^^,^^9^^,^^10^ With the growth of primary TKA in absolute terms, it is reckoned that the frequency of revision TKA (RTKA) surgery will also increase.^11^

The growth rate of TKA surgery has tended to track the progressive aging of the population and, moreover, the recommendations relating to the operative procedure have been extended to bring in patients who, increasingly, are younger and more active.^12^^,^^13^ On the other hand, obesity continues to increase and can be considered to be a risk factor for TKA and for complications after TKA.^9^^,^^11^ The cost of the prosthetic implants is one of the main expenses relating to the charge of knee surgery procedures, and the average price for prosthetic knee implants continues to follow the upward trend seen over the last decade.^11^^,^^14^

The increasing prevalence of RTKA surgery is related to several factors. Among the most notable of these are the additions to the indications for TKA; factors associated with poor surgical technique and use of inappropriate instruments; incorrect choice of patients; the longevity of prosthetic knees; and occurrence of infection.^12^^,^^13^ Thus, recent predictions point towards a substantial rise in the number of RTKA procedures over the next few decades.^12^^,^^15^

In Scandinavian countries, the increase in the number of TKA and RTKA procedures has raised the level of healthcare spending through the increased length of time for which patients are hospitalized, higher cost of implants and additional morbidity.^15^^,^^16^ In order to better allocate financial resources and ensure efficient hospital management and implementation of economic and educational policies, it has been recommended that research should be conducted on epidemiological data relating to TKA and RTKA procedures.^15^^,^^16^^,^^17^^,^^18^^,^^19^ In developing countries, few studies have assessed such data.^20^^,^^21^

## OBJECTIVE

The aim of this study was to estimate the frequency of knee arthroplasty procedures carried out by the public healthcare service of the state of São Paulo between 2003 and 2010. 

## METHODS

This study was conducted by researchers at the Orthopedics and Traumatology Service of the Hospital do Servidor Público do Estado de São Paulo, with the approval of this institution's ethics committee (protocol no. CAAE 34035314.1.0000.5463).

Data on epidemiological assessments of knee arthroplasty procedures carried out in the whole state of São Paulo between January 2003 and December 2010 were gathered from the TABNET^2^ and SIGTAP^22^ databases (management systems for the table of procedures and medications and for the personnel management office of the public healthcare service). These databases are managed through the public healthcare service's hospital information system (Sistema de Informações Hospitalares/Sistema Único de Saúde, SIH/SUS) and are overseen by the Ministry of Health through the Department of Healthcare, together with state health departments and municipal health departments. The data are processed by the public healthcare service's information technology department (Departamento de Informática do Sistema Único de Saúde, DATASUS), which is part of the Ministry of Health. Patients are registered for inclusion in this database through the hospital admission authorizations that are issued for patients who are admitted with the condition in question, in accordance with the description under the International Classification of Diseases, 10^th^ revision (ICD-10).

The disease codes used for the diagnosis of OA were included in the ICD-10 category M17. The hospitals included in this study are listed in Appendix 1. The procedure codes used in the National Hospital Discharge Survey (NHDS) for identifying TKA and RTKA were 04.08.05.006-3 and 04.08.05.005-5, respectively.

Patients who underwent TKA and/or RTKA were assessed. All of these patients were registered in the TABNET software. Partial arthroplasty procedures (unicompartmental and patellofemoral) were excluded.

### Statistical analysis

The prevalence of knee arthroplasty procedures was estimated for each gender, as the number of primary procedures (TKAs) and number of revisions (RTKAs). The variables assessed are presented in tables showing absolute and relative frequencies. The normality of the variables was tested using the Shapiro-Wilk test, and the ratios of the variables were compared using a test for the equality of two proportions.^21^ Trend analysis was carried out using polynomial regression models. These models were chosen for their high power, from a statistical perspective, and for the ease with which they can be created and interpreted.^23^

All of the analyses were conducted using a significance level of 5%. The alternatives of two-tailed hypotheses were always taken into consideration.

The information gathered was compiled into a database using Excel for Windows and the statistical analysis was carried out using the STATA 11 SE and Minitab 16 software.

## RESULTS

The total number of knee arthroplasty procedures carried out in the study period in the state of São Paulo was 10,952 (7,891 females and 3,061 males), with an annual average of 1,369 (ranging from 921 to 2,259) and a standard deviation of 481.

The total number of TKA procedures was 9,271, with an annual average of 1,159 (ranging from 830 to 1,839) and a standard deviation of 361. The ratio of TKA procedures to RTKA procedures was 85%, which was statistically significant (P < 0.0001), i.e. 5.5 times as many TKA procedures as RTKA ones were carried out (Table 1).

**Table 1:**
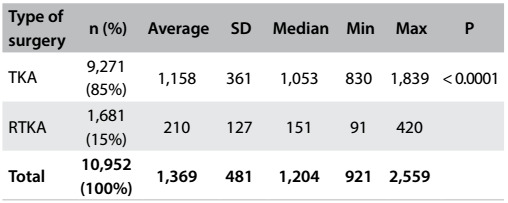
Distribution of primary total knee arthroplasty (TKA) and revision total knee arthroplasty (RTKA) procedures from 2003 to 2010

**Figure 1:**
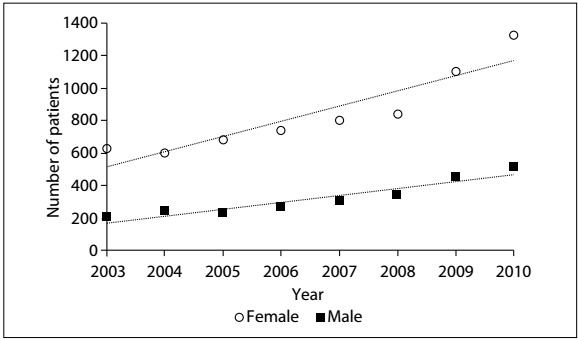
Trends in primary total knee arthroplasty (TKA) procedures according to gender.

Analysis on the knee arthroplasty data (Table 2) showed that the number of female cases (72%) was significantly greater than the number of male cases. This difference was statistically significant (P < 0.0001). This gender bias was maintained with regard to comparing the data relating to TKA. It could be seen that the numbers of TKA procedures on both females and males is rising (both with P < 0.001) and that 2.6 times as many procedures are carried out on females. The growth trend for the female population is more pronounced than that of the male population. In 2003, 627 TKA procedures were carried out on females and 203 on males. In 2010, 1,325 TKA procedures were carried out on females and 514 on males. This represents an increase of 133% for women and 153% for men (Figure 1).

**Table 2:**
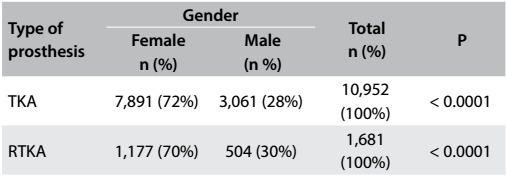
Distribution of revision total knee arthroplasty (RTKA) and total knee arthroplasty (TKA) procedures according to gender between 2003 and 2010

A total of 1,681 RTKA procedures were carried out, with an annual average of 210 (ranging from 91 to 420) and a standard deviation of 127. A significantly greater proportion of the procedures (70%) were conducted on females than on males (P < 0.0001) (Table 2).

**Figure 2:**
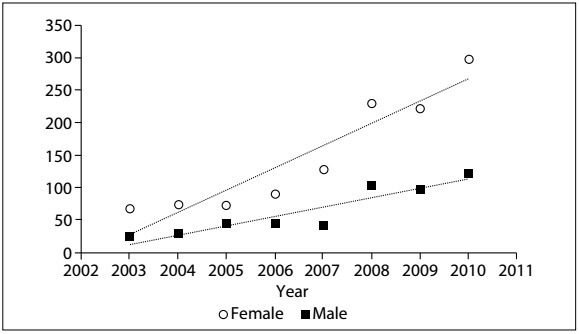
Distribution of revision total knee arthroplasties (RTKA) according to gender between 2003 and 2010.

Figure 2 shows that there was a rising trend in the number of RTKA procedures carried out, for both males and females (both at P < 0.001), but that a greater proportion of the procedures were carried out on females, with an average of 2.3 procedures on females for every procedure on a male, which is in keeping with the ratio for TKA. In 2003, there were 67 TKA procedures carried out on women and 24 on men, while in 2010 the numbers were 298 and 122 respectively. This represents growth of 344% for women and 408% for men.

**Figure 3:**
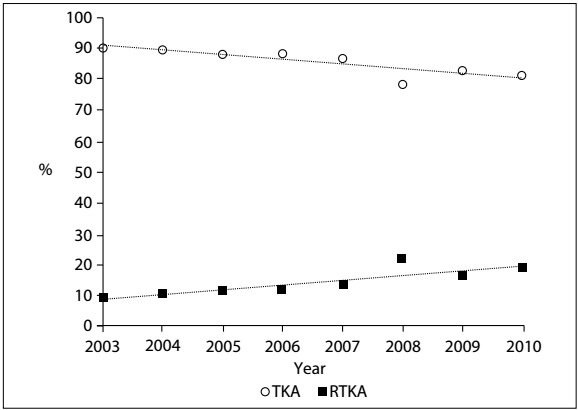
Ratio of total knee arthroplasty (TKA) to revision total arthroplasty (RTKA) procedures between 2003 and 2010.

It can be seen that the number of TKA procedures is growing in absolute terms (Table 1). However, the ratio of primary TKA to revision TKA procedures has fallen year on year, as shown in Figure 3. In 2003, there was one revision operation for every nine primary operations, whereas in 2010, this ratio fell to one RTKA for every 4.4 TKA procedures.

## DISCUSSION

In analyzing the data provided by DATASUS, it can be seen that between 2003 and 2010 there was a change in the epidemiological profile of the knee arthroplasty procedures carried out in the state of São Paulo. There was an increase of 334% in the number of revision TKA procedures carried out on females during the period observed. This increase was significantly greater than in another study in the United States, in which the increase for females was 30%.^24^ Our gender-specific analysis on TKA showed that there was an increase of 111% for women, while some similar studies conducted in other countries found proportions of 67%,^24^ 74.7%^25^ and 90%.^26^ This difference may be due to the greater proportion of females in the elderly population and the relatively lower acceptability of TKA among male patients for cultural reasons.

There was an increase in the absolute number of prosthetic knees used. The number of TKA procedures (rise of 122%) more than doubled and the number of RTKA procedures (362%) virtually quadrupled. Other studies, covering similar periods of time, resulted in figures that predicted proportional growth between the two types of surgery, in the United States (140% TKA and 166% RTKA),^24^ in Taiwan (99.1% TKA and RTKA 138%)^25^ and in Korea (407% TKA and 267% RTKA).^26^ A further American survey showed linear progression between TKA and RTKA between 1990 and 2002 (both tripled over that period), with the ratio changing from 10.75 to 10.88.^24^ Our series showed a decrease in this relationship between 2003 and 2010, such that the ratio changed from 9.0 to 4.4. This trend differed from the typical ratio of 11 primary TKA procedures for each revision TKA procedure, seen in other studies.^25^^,^^26^ In our sample, this proportion was not as marked. This may have resulted from the increase in the number of applications for TKA, which has contributed towards raising the RTKA rates.

The most common cause of RTKA is infection.^13^^,^^14^^,^^15^ Preventive measures have been recommended in order to preclude infection after TKA, such as: laminar flow in the operating room, body exhaust suits for the surgical team, waterproof paper drapes, waterproof gowns, double-gloving with outer glove changes after draping and at regular intervals during surgery, skin preparation with 2% chlorhexidine plus 70% alcohol, usage of antibiotic-loaded cements under some circumstances (diabetes, rheumatoid diseases, smokers, previous surgery, malnutrition or coagulopathy), appropriate dosage and choice of systemic prophylactic antibiotics and reduction of allogenic blood transfusion, so as to avoid routine use of surgical drains and bladder catheters, and to minimize the duration of the operation and the number of people circulating during the procedure.^27^ These measures have not been adopted routinely in the hospitals analyzed in other countries^16^^,^^24^^,^^25^^,^^26^ and this may be a possible reason for the increase in the RTKA rate between 2003 and 2010.

This expansion in both types of arthroplasty can be explained by improvements to instruments and prosthetic implants and improved surgical techniques over recent decades, as well as by the good and sometimes excellent results achieved over the long term among patients who have undergone TKA and RTKA.^9^^,^^14^^,^^19^^,^^28^ Another factor that helps explain this progress is the relationship between obesity and knee osteoarthritis,^29^^,^^30^ in the light of the increasing prevalence of obesity among the populations of Western countries that has been observed over the last decade.^29^^,^^30^^,^^31^^,^^32^ The present study did not cover this specific variable, nor did it cover other risk factors involved in recommendations for knee arthroplasty surgery in the sample that was evaluated.

This spread of TKA will result in a call for improvements in hospital management and extra training for medical professionals and nursing teams, given that there is a positive association between surgeons' experience and the volume of surgical procedures. Thus, the leading surgical centers that specialize in joint replacements have the highest success rates from knee arthroplasty.^10^^,^^31^^,^^33^ This may have been a factor that contributed towards a higher rate of RTKA. Well-trained knee surgeons who have specialized in TKA and RTKA for one year after their fellowship report fewer complications than do non-specialized surgeons.^34^ In the present study, the level of surgeon expertise in knee arthroplasty was not evaluated and may have been a source of poor outcomes from TKA due to technical errors, thereby increasing the RTKA rate.

These numbers are unlikely to be sustained over the long term, given that new materials and more advanced techniques, including those used by surgeons for implanting primary prostheses, will tend to lower the numbers of RTKA procedures.^10^^,^^14^^,^^35^ Nonetheless, in developed countries, the RTKA rate has increased over time,^24^^,^^26^ even in situations in which the public healthcare system predominates.^25^^,^^36^

The weaknesses of the present study include: the lack of standardization in surgical techniques; the absence of data covering elements such as the sociodemographic variables of age, education level and income, along with patient comorbidities (including obesity), which could help identify risk factors for knee arthroplasty; the absence of the osteoarthritis etiologies and recommendations for the knee arthroplasty procedures performed; and the lack of knowledge relating to materials and implants used for TKA and RTKA. These data were not gathered because of the diversity of information in the medical records and because no interviews with the patients who underwent surgery could be conducted.

## CONCLUSION

The number and prevalence of TKA and RTKA procedures increased in the state of São Paulo during the period between 2003 and 2010. Proportionally, this rise was greater in relation to RTKA than to TKA. This increase was more prominent among female patients.
